# Functional 3-Dimensional Retinal Organoids: *Technological Progress* and Existing Challenges

**DOI:** 10.3389/fnins.2021.668857

**Published:** 2021-04-20

**Authors:** Meimanat Fathi, Cody T. Ross, Zohreh Hosseinzadeh

**Affiliations:** ^1^Department of Cell Techniques and Applied Stem Cell Biology, Faculty of Medicine, Center for Biotechnology and Biomedicine (BBZ), University of Leipzig, Leipzig, Germany; ^2^Physiology and Pathophysiology of the Retina Group, Department of Molecular and Cellular Mechanisms of Neurodegeneration, Paul Flechsig Institute of Brain Research, University of Leipzig, Leipzig, Germany; ^3^Department of Human Behavior, Ecology and Culture, Max Planck Institute for Evolutionary Anthropology, Leipzig, Germany

**Keywords:** organoids, retina, functionality, visual cycle, ON/OFF pathways

## Abstract

Stem cell scientists have developed methods for the self-formation of artificial organs, often referred to as organoids. Organoids can be used as model systems for research in multiple biological disciplines. Yoshiki Sasai’s innovation for deriving mammalian retinal tissue from *in vitro* stem cells has had a large impact on the study of the biology of vision. New developments in retinal organoid technology provide avenues for *in vitro* models of human retinal diseases, studies of pathological mechanisms, and development of therapies for retinal degeneration, including electronic retinal implants and gene therapy. Moreover, these innovations have played key roles in establishing models for large-scale drug screening, studying the stages of retinal development, and providing a human model for personalized therapeutic approaches, like cell transplants to replace degenerated retinal cells. Here, we first discuss the importance of human retinal organoids to the biomedical sciences. Then, we review various functional features of retinal organoids that have been developed. Finally, we highlight the current limitations of retinal organoid technologies.

## Introduction

The retina is a thin (∼0.25 mm) layer of neurons in the back of the eyeball—it is a part of the central nervous system that grows inside of the eye during development. In the nineteenth century, Santiago Ramon y Cajal, father of the neuron theory of the nervous system, introduced the first comprehensive morphology of neural cell types, including the cells of the retina, in a number of vertebrate species (Lerma and De Carlos, 2014). Since then, many studies have investigated different aspects of this complex system and its development through ontogeny. We now know that the retina is composed of several layers, with different cell types consisting of horizontal, bipolar (BCs), amacrine, and ganglion cells (GCs), as well as photoreceptors (PRs), and three types of glial cells, including microglia, astrocytes, and Müller glial cells (Bringmann et al., 2006). However, many features of vertebrate retinal function and development are still unknown, making it difficult to recapitulate the retina *in vitro* using stem cells.

**FIGURE 1:**
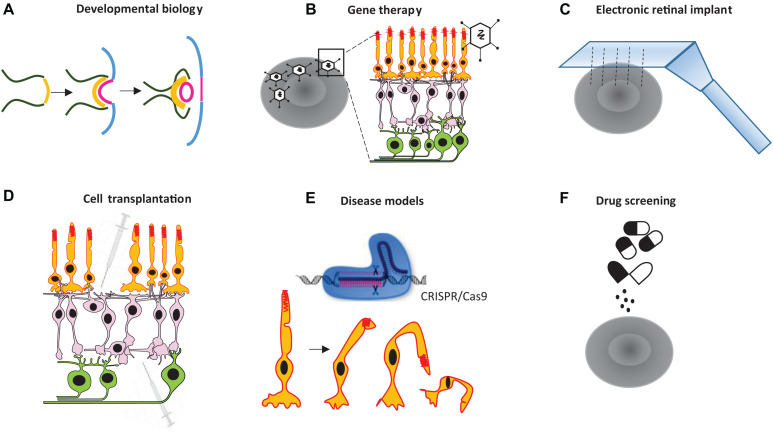
Some potential applications for retinal organoid technology. Retinal organoids can be used to **(A)** study the development of the retina, **(B)** investigate various therapeutical approaches, including gene therapy for retinal disorders, **(C)** test electronic chip implants, **(D)** facilitate cell transplantation, **(E)** test human retinal disease models, and **(F)** conduct drug screening.

Organoids derived from induced pluripotent stem cells (iPSCs), or embryonic stem cells (ESCs), have been used by researchers to create *in vitro* tissues that mimic their natural counterparts, advancing medical research in the 1980s and beyond (Shannon et al., 1987). More recently, new methodological advances for culturing tissues have opened up new possibilities for basic research on various human organs—e.g., the brain (Qian et al., 2019), intestine (Sprangers et al., 2020), kidney (Taguchi et al., 2014), prostate (Gao et al., 2014), and retina (Zhong et al., 2014; Cowan et al., 2020). Scientists have successfully developed retinal organoids that closely resemble many aspects of the real retina using human and mouse stem cells. Lab-grown retinal organoids are composed of several types of cells organized in a physiologically and morphologically complex manner (Eiraku et al., 2011). A retina-specific synapse, referred to as the ribbon synapse, is formed in such organoids (Castro et al., 2019). These organoids also show physiological responses to light stimuli to some degree (Zhong et al., 2014; Wahlin et al., 2017). As such, retinal organoids can be used as a basic model for investigating various therapies or treatments. For example, retinal organoids can be used to study retinal degeneration, human retinal implants, optogenetics and gene therapies, drug screening and toxicity, and the stages of retinal development. Therefore, they provide a human model for personalized therapeutic approaches and can be used in transplants of a patient’s degenerated retinal cells (see the summary of potential applications of retinal organoids in Figure 1). Despite recent progress in retinal organoid technology, our knowledge is still in its infancy, and organoids have not recapitulated all developmental stages of the natural retina.

In this review, we discuss some real-world problems illustrating the necessity of speeding up the development of retinal organoid technology for medical research. We then highlight the key functional aspects of retinal organoids—e.g., visual cycles, synaptogenesis, and retinal pathways—and the physiological recovery of the retina after retinal cell or organoid transplantation. Finally, we discuss current constraints regarding, and unanswered questions about, retinal organoids.

## The Necessity of Improving Retinal Organoid Models

According to the World Health Organization [WHO] (2019), more than one billion people globally have a vision impairment that could have been prevented or is yet to be addressed. According to eye health data and statistics, the number of people with the most common eye diseases will double by 2050 (Varma et al., 2016). In order to better understand retinal diseases, and develop treatments for them, a proper retinal model is critically needed.

Donated post-mortem retinal tissues, especially those affected by retinal disorders, are suitable *in vitro* testbeds for investigating underlying pathophysiology mechanisms. The quality of data obtained from the molecular biology, the imaging, and the visual function of a post-mortem retina depends on rapid isolation and a regular oxygen supply (Pannicke et al., 2005; Cowan et al., 2020). Gene expression can change rapidly post-mortem in a tissue-specific manner (Ferreira et al., 2018). Post-mortem transplantation of eye components depends on the persistence of tissue; for example, 36% of corneal transplants using post-mortem tissues with death-to-preservation times of more than 6 h failed (Mohamed et al., 2016). There are several constraints on using donated post-mortem retinal tissues, including organ availability and its pathological state, as well as the ethical requirements of the state and institution where the procedure is conducted.

The 2-dimensional (2D) culture of immortalized retinal cell lines is a resource for study of retinal pathologies and drug testing. 2D culture of different retinal cell lines is needed for studying different retinal diseases. For example, human retinal pigment epithelium (RPE) cell lines can be used to study age-related macular degeneration (AMD) (Kozlowski, 2015), and MIO-M1 human Müller glial cell lines can be used to study diabetic retinopathy or retinal degeneration (Yong et al., 2010). Moreover, the 2D culture of reprogrammed iPSCs-derived retinal cells (Lamba et al., 2006) has been established for the study of multiple conditions—e.g., disease modeling, drug testing and discovery, and cell replacement therapies (O’Hara-Wright and Gonzalez-Cordero, 2020). Nevertheless, 2D retinal culture may not emulate important naturalistic aspects of native retinal cells *in vivo*, and also fails to entirely recapitulate the morphology and functional physiology of the human retina.

Animal models are another widespread tool that can be used to study retinal diseases and develop treatments—e.g., through genetic or interventional modifications (Volobueva et al., 2019). The retinal tissue of non-human species can mimic aspects of retinal diseases found in humans. For example, rd10 mice carrying a spontaneous mutation of the phosphodiesterase gene in rod cells are a model of autosomal recessive retinitis pigmentosa, which leads to rod degeneration in humans (Gargini et al., 2007). Animal models assist researchers in understanding diseases and their prevention, diagnosis, and treatment, in cases where human research would be impractical or ethically prohibited. Although our understanding of retinal pathophysiology has been improved by animal models, there are considerable drawbacks in extrapolating the findings of rodents to humans. For example, the absence of the macula and photopigmentation in rodents, differences in color vision—e.g., dichromatism in rodents (Szatko et al., 2020) vs. trichromatism in humans—and differences in tissue structure, size, refractive properties, and the nature of the retinal vascular system (Achberger et al., 2019b; Castro et al., 2019). A key solution for overcoming the aforementioned limitations would be to generate 3-dimensional (3D) functional retinal tissues *in vitro*.

## Retinal Organoid Functionality

### The Visual Cycle in Retinal Organoids

Retinal pigment epithelium (RPE) plays a critical role in photoreceptor survival and functionality, as well as modulation of the visual cycle—the conversion of an incoming photon into an electrical signal (Wright et al., 2015). Thus, in order to generate functional retinal organoids, it is important to integrate components of the visual cycle, including RPE. RPE is composed of polarized cells within the basal and apical membranes. On the apical side, RPE connects with PRs through a network of microvilli, which allows exchange of materials between PRs and RPE (Kang et al., 2009; Sahu and Maeda, 2018). RPE supports PRs by providing nutrients, removing waste products, releasing growth factors, and regulating the length of PRs by phagocytosis. Most importantly, the RPE plays a fundamental role in retinol cycling by recycling 11-cis retinal from all-trans isomers for the next visual cycle (Ramsden et al., 2013; Tsin et al., 2018; Choi et al., 2020). The expression of RPE markers includes MERTK (a phagocytosis marker) and BEST1 (a basal marker), as well as RPE65, LRAT, and CRALBP (which are visual cycle markers). Each of these markers has been identified *in vitro* in RPE differentiated from iPSCs (Chichagova et al., 2018).

RPE spheroids isolated from hiPSC-derived retinal organoids are able to reach maturation. Such a state of cellular, molecular, and physiological maturity is indicated by a number of features, including repigmentation, marker expression, and phagocytosis (Liu et al., 2018). Co-culturing retinal organoids with hiPSC-derived RPE elevates the differentiation of retinal progenitors derived from human iPSCs (Akhtar et al., 2019), suggesting that RPE promotes retinal differentiation. In the presence of 9-cis retinal, rod differentiation is accelerated in organoid cultures with proper rhodopsin expression at day 120, indicating visual cycle control between RPE and PRs as an essential part of retinal development (Kaya et al., 2019). Maturation of PRs is promoted in the presence of retinoic acid (the pleiotropic all-trans-retinol form) (Zerti et al., 2020). To date, the co-culturing of RPE with supplementary components, like 9-cis retinal, is the only available method for activating the visual cycle (to some degree) in retinal organoids. We discuss this further in the section: “Current limitations of retinal organoid technology.”

### The Maturation of Retinal Pathways

Visual inputs to the retina are encoded by distinct visual pathways in parallel for transmission to the brain via ganglion cells (GC). In mice, there are approximately 15–20 channels (pathways) based on GC dendritic morphologies (Coombs et al., 2006; Volgyi et al., 2009) and 30 functional output channels based on calcium imaging recordings (Baden et al., 2016). ON and OFF channels, which are responsible for the fundamental functional features of the visual system, can be recorded by electrophysiology techniques (Popova, 2014). ON pathways (ON cells) are activated by light increments, whereas OFF pathways (OFF cells) are activated by light decrements. The characterization of ON and OFF pathways is one of the main goals in retinal organoid engineering.

Single cell patch clamp recording lets us identify hyperpolarization and depolarization of mature PRs in retinal organoids, responding to light and darkness, respectively. Zhong et al. (2014) reported, by means of the patch clamp technique, that 2 out of 13 randomly chosen PRs responded to a light stimulus in hiPSC-derived retinal organoids at weeks 25–27. The authors inferred that the low number of responsive PRs could be due to the low levels of rhodopsin and downstream phototransduction steps that may still be under maturation (Zhong et al., 2014). Cones targeted by patch clamp recording reveal the presence of hyperpolarization-activated cyclic nucleotide-gated (HCN) channels, the fundamental key of phototransduction in PRs (Kim et al., 2019).

Using a microelectrode array (MEA), one can record the action potential of GCs in order to identify network-mediated retinal pathways. The response of GCs in retinal organoids to light stimulus has been recorded at day 150 by MEA. These responses, however, were too indolent to document clear matched spikes to light pulses (Hallam et al., 2018). Such an event might be comparable with spontaneous waves of activity sweeping across the neonatal vertebrate retina before eye-opening (Maccione et al., 2014). Puffing 8-br-cGMP, a secondary messenger of the phototransduction cascade, binding to the gates of Na^+^ -permeable channels, depolarizes PRs, thus mimicking dark current. In the presence of 8-br-cGMP puff in organoid cultures, MEA recording suggests that GCs with decreased spiking activity are ON, whereas GCs with increased firing rate are OFF (Hallam et al., 2018). The decellularized extracellular matrix-derived peptides from the neural retina (decel NR) and RPE conditions of retinal organoid cultures promote the light-driven responses of ON RGCs of organoids recorded by MEA (Dorgau et al., 2019). Very recently, Cowan et al. (2020) reported the transmission of light responses through the cascade of PRs to BCs and GCs in organoids by using live two-photon laser imaging; however, the percentage of the responsive cells was low (Cowan et al., 2020). To some extent, the ON and OFF retinal pathways of organoids have been characterized, but such responses are still far from replicating the spike activities of ON and OFF pathways *in vivo*. We discuss this further in the section: “Current limitations of retinal organoid technology.”

### Synaptogenesis in Retinal Organoids

The retina processes different visual features in parallel—e.g., brightness, darkness, color, contrast, and motion (Wassle, 2004). For the processing of visual signals, synapses with different kinetics in signal transmission are needed. Indeed, the retina possesses a range of synapses, including chemical, fast electrical, and ribbon synapses.

The somas of retinal neurons are located in the three nuclear layers of the retina, which are separated by a minimum of two synaptic layers. Synapses transmit the signals at the tonic rate of transmitter release and in a graded fashion. Two ribbon synapses exist in the retina: PRs transmit their signals in the outer plexiform layer (OPL), and BCs send signals in the inner plexiform layer (IPL). The ribbon is poised for fast transmitter release, and is ideal for reporting the rapid onset of stimuli (tom Dieck and Brandstatter, 2006; Lagnado and Schmitz, 2015; Okawa et al., 2019). The generation of such synaptic ribbon has been reported during retinal organoid development (Cowan et al., 2020; Sridhar et al., 2020). In human retinal organoids, PR ribbon synapses with vesicles and close contact with BCs and HCs have been observed (Cowan et al., 2020). Presynaptic ribbon markers (RIBEYE and SYNTAXIN3) contain vesicles, close to dense bars, in the synaptic endfeet of the OPL-like region (Gonzalez-Cordero et al., 2017). Moreover, the interconnection between PR axons and BC dendrites forming the retinal-specific ribbon synapses in retinal organoids has been observed using the passive clearing technique (Cora et al., 2019). Ribbon synapses at BCs in the IPL of retinal organoids have not yet been identified. To our knowledge, ribbon synapses at rods and cones of retinal organoids have not been distinguished either.

## Visual Function and Cell Transplantation

### Visual Function After Cell Transplantation

Cell transplantation is a novel therapeutic strategy to restore visual responses that have been lost to retinal degeneration or disease—for example, inherited retinitis pigmentosa or AMD (West et al., 2009; Stern et al., 2018). Stem-cell-derived retinal organoids and RPE organoid therapy have great therapeutic potential for treating such degenerative diseases. In this section, we focus on visual function after transplanting organoids and RPE sheets or cells isolated from them.

### Photoreceptor Transplantation

Transplanted purified human cones isolated from retinal organoids (combining 2D/3D methods) have been incorporated within Nrl^–/–^ mouse retinas with defective S- and M-cone opsins (Gonzalez-Cordero et al., 2017). Subretinally transplanted 3D hESC-derived retinal organoid sheets (day 30–65) in rho S334ter-3 rats have differentiated, integrated, and produced functional PRs, resulting in visual function and active synapses in the recipients’ eyes (McLelland et al., 2018). Jaws-positive PRs (with red-shifted cruxhalorhodopsin) isolated from retinal organoids and subretinally transplanted into blind hosts (mice) can become integrated into the host retina and ON- and OFF pathway responses (Garita-Hernandez et al., 2019).

Cell transplantation may lead to natural interactions among donor and host cells, and transmit cytoplasmic materials. Numerous observations indicate that functional recovery of the host retina might be achieved by transferring cytoplasmic material from transplanted PRs to remaining host PRs (Santos-Ferreira et al., 2016; Chichagova et al., 2018; Gagliardi et al., 2018). Nevertheless, cell transplantation could lead to natural interaction of donor and host cells, as well as transmission of cytoplasmic materials. Transplanting hESC-derived retinal organoids into the retinas of immunodeficient RCS nude rats has been shown to improve visual function; transplanted cells migrate and integrate synaptic connectivity with the host cells during the transfer of cytoplasmic material (Lin et al., 2020).

### RPE Transplantation

The transplantation of hESCs-derived RPE cells in patients with AMD has shown promising outcomes in clinical trials (phase I/II)—such outcomes include increases in vision-related quality-of-life and improvements in best-corrected visual acuity (but only in some patients) (Schwartz et al., 2015). Later, Takahashi’s group reported that hiPSC-derived RPE sheets transplanted into human retinas with AMD remained intact and showed good retinal integrity, unchanging visual acuity, and no immune rejection (Mandai et al., 2017).

Cell transplantation entails several challenges: obtaining high-quality transplantable cells in sufficient quantities, achieving integration and function of transplanted cells (Gonzalez-Cordero et al., 2017), and minimizing the risk of cancerogenesis (Singh et al., 2018). Finding specific markers of retinal cells for purification may provide a solution that improves the efficiency of obtaining cells. For example, Gagliardi et al. (2018) has developed the CD73 + PRs MACS method to purify and sort a pool of PRs from hiPSC-derived retinal organoids for transplanting into a host retina (Gagliardi et al., 2018). The other shortcomings remain to be resolved.

## Current Limitations of Retinal Organoid Technology

**FIGURE 2:**
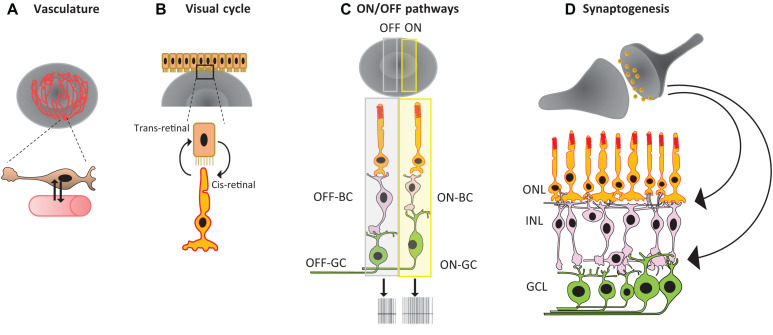
Summary of major challenges in retinal organoid technology: **(A)** lack of vasculature, **(B)** RPE orientation for the visual cycle, **(C)** mature ON and OFF pathways, and **(D)** proper cell orientation and synaptogenesis.

Although the field of retinal organoid technology has seen significant progress in recent years, the construction of highly complex mammalian retinas *in vitro* is still beyond the reach of our current tools. It is still not possible to generate retinal organoids that have the same biochemical and physiological characteristics as mature *in vivo* retinas. In order to create such organoids, future technology must integrate several additional features, including smooth muscle cells, vasculature, and immune cells like microglia. Additionally, researchers must ensure proper cell orientation and wiring within retinal neurons, and correct RPE orientation for the maturation of the visual cycle and mature ON and OFF pathways, as we discuss in more detail below (see Figure 2, and Achberger et al., 2019a; Kruczek and Swaroop, 2020; Singh and Nasonkin, 2020).

### Vasculature

One of the major challenges in producing 3D organoids is the maintenance of long-term cell viability, which strongly depends on access to nutrients and oxygen (Ozbolat, 2015). The current inability to supply such nutrients has been attributed to the lack of endogenous, engineered vasculature or nutrient channels in the organ (Laschke and Menger, 2012). Without vasculature, the size of an organoid is limited to the diffusional limit of oxygen (Radisic et al., 2006). This problem may be resolved by applying new technological approaches—for example, optimization of scaffold porosity (Chen et al., 2019), inclusion of bioreactor (Ovando-Roche et al., 2018), incorporation of oxygen delivery mechanisms (Li et al., 2012), retina-on-a-chip (Achberger et al., 2019b), 3D bioprinting of vascularized tissues (Richards et al., 2017), and co-culture with mesodermal progenitor cells (Worsdorfer et al., 2019) are areas of current research.

### Microglia

Microglial cells are primary resident immune cells that interact with Müller glial cells in the retina. Microglial cells are important for the natural development of a retina because they regulate neuronal survival and synaptic pruning (Li et al., 2012). Microglial cells and other retinal cells in organoids do not arise under the same differentiation conditions, due to differences in their lineages. Microglial cells come from the hematopoietic lineage that colonizes brain tissue during embryonic development (Cunningham et al., 2013). It is suspected that the existence of microglia within the retina may be key to successful retinal development (Li et al., 2019).

### RPE

In retinal organoid cultures, mature and functional RPE around PRs is missing. RPE is a retinal cell type that is essential for retinal development. An ablation of RPE in mouse retina at embryonic day E10–11 leads to disruption of the retinal layers and eye growth. This deficiency does not affect retinogenesis at embryonic day E11.5–12.5; however, it disrupts the laminar structure and vitreous production (Raymond and Jackson, 1995; Fuhrmann et al., 2014). Indeed, RPE plays a vital role in the protection and survival of PRs (German et al., 2008). This is confirmed by the fact that the differentiation of PRs has been improved by co-culturing the retinal organoid with RPE (Akhtar et al., 2019). RPE maintains the physiology of the outer retina—for example, by enacting phagocytosis of shed PR outer segments, running the visual cycle, and secreting neurotrophic and vasculotrophic growth factors that promote vascularization (Sparrow et al., 2010; Achberger et al., 2019b). Therefore, the interaction between the retina and RPE is critical, not only during retinal organoid development, but also for visual function in a mature retina.

### Degeneration and Loss of Orientation

Another major limitation in producing 3D retinal organoids is degeneration and the loss of orientation through development. The maturation of human retinal organoids *in vitro* proceeds at about the same rate as human retinal development *in vivo* (Nakano et al., 2012; Zhong et al., 2014; Wahlin et al., 2017). This long developmental process leads to the degeneration of the organoid. Such degeneration is generally caused by a lack of nutrition or poor passive diffusion, mainly in the inner layers. Therefore, the inner cells disappear before the PRs reach a fully mature stage in the organoid (Reichman et al., 2014). In contrast to the natural cup shape of the retina *in vivo*, retinal organoids have a spherical form. This shape may be caused by a tendency of suspended cells to form into spheroids with an equal distribution of surrounding cellular layers (Forgacs et al., 1998; Ajduk and Zernicka-Goetz, 2016). Lacking orientation, surrounding tissue and extracellular matrix prevent a proper polarized tissue development (Takano et al., 2015), which is necessary for optic-cup development and peripheral-central specialization.

### Synaptogenesis

Retinal synapses are organized in the inner and outer plexiform layers in an orderly fashion. This structure can efficiently process visual signals—for example, through horizontal and vertical synaptic pathways (Wassle, 2004). Immunostaining and electron microscopy have shown ribbon synapse expression in retinal organoids, which in turn may conduct signals from the PRs to the BCs (Wahlin et al., 2017). However, there is a lack of sophisticated traits in the outer plexiform synapses (Haverkamp et al., 2000) within horizontal cells, PRs, and BCs in retinal organoids. Currently, ribbon synapses between BCs, amacrine cells, and GCs have not been detected in retinal organoids. This finding could be due to the disappearance of the inner layer, including the GCs after a certain time period in the organoid culture process—e.g., before maturation of the PRs (Reichman et al., 2014; Zhong et al., 2014). Because BCs arise late in development (Cepko, 2014), GCs may have already disappeared. Moreover, rod and cone ribbon synapses have not been discovered in retinal organoids. Nevertheless, niches like IGF-1 for wiring between retinal cells may influence the formation of synapses in the inner layer of retinal organoids (Mellough et al., 2015).

### Retinal Pathways

**FIGURE 3:**
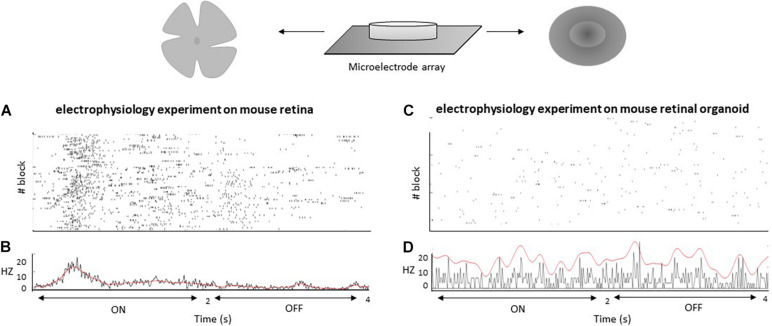
MEA recordings from mouse retina at day P1 **(A)** and mouse retinal organoid at day 22 **(C)**. Frames **(A,C)** provide rastergrams of all responses for cells to visual stimulation blocks with a stimulus of 2 s ON and 2 s OFF that was cycled 25 times per block (3 blocks). Frames **(B,D)** provide peristimulus time histograms for all responses.

A fundamental functional feature of the retina is its ability to discriminate between different light stimuli in a way that is recordable by electrophysiology. This ability results from the fact that the retina is composed of different cell types with complex connectivity. However, comparing *in vitro* retinal organoids to *in vivo* retinal tissue, researchers have found that retinal organoids are far less morphologically and functionally sophisticated in terms of their synapses, connectivity, and cell subtype varieties (Masland, 2012). Even in long-term cultures, retinal organoids are incapable of generating and maintaining the three clearly separated nuclear layers found in *in vivo* retinas (Dorgau et al., 2019). Because of this fact, retinal organoids generally lack the complex arrangement of rods and cones found in natural retinas, as well as the connections from rods and cones to interneurons and GC types—e.g., ON and OFF pathways, direction-selective pathways, etc. (Baden et al., 2016; Behrens et al., 2016). Retinal organoids are not only unable to completely respond to light stimuli, but they are also characterized by a lack of retinal pathways, particularly ON and OFF pathways (Dorgau et al., 2019). Immature outer segments of PRs, as initial components of photocascades, might be one of the reasons (Castro et al., 2019). Using MEA as a method to measure retinal pathways, researchers have shown that GCs in organoids are responsive to light stimuli; however, these responses are indolent and can be hard to separate from background noise and spontaneous spikes (Hallam et al., 2018). This behavior is similar to spontaneous activities of the retina during embryonic time (Maccione et al., 2014). However, light responses are clearly detectable at the same age in mice (Ford et al., 2012). New protocols for generating retinal organoids have partially improved responses to light, as measured by MEA recordings (Dorgau et al., 2019); however, such recordings are not comparable with clear light responses *in vivo*. Using calcium imaging with a two-photon laser microscope, only 16.7% of cells in the outer nuclear layer and 12.0% of cells in the inner nuclear layer of retinal organoids are light-mediated responsive (Cowan et al., 2020), reflecting immature retinal pathways. It appears that retinal organoids vary to some extent in their structure and functionality, a fact that might emanate from various properties of iPSCs lines (Castro et al., 2019). Hosseinzadeh and Fathi (2021) recorded ON and OFF light responses in mouse retinal organoids at day 22 and mouse retinas at postnatal day 1 (P1) via MEA. Mice retinal organoids have not shown any clear responses to light modulation (ON and OFF pathways), while mouse retinas have responded to such stimuli (see Figure 3). However, mouse retinal organoids have responded to electrical stimulus pulses. In addition, retinal organoids have generated spontaneous action potentials, as shown in Figure 3. Overall, retinal organoids mimic the earlier fetal stage concerning electrical activities, with some signs of light responses, but a lack of the clear character of two main retinal pathways, a shortcoming waiting to be resolved. Solutions for this shortcoming are likely to be closely related to synaptogenesis and establishment of electrical retinal pathways.

## Conclusion

It is an exciting time to generate 3D organs *in vitro.* Technological advances have made much possible; however, several challenges remain to be resolved. ESCs- and iPSCs-derived cell subtypes play a promising role in the generation of retinal organoids, and have clinical implications, especially in personalized medicine. Overall, hiPSCs-derived retinal organoids are an advanced tool for overcoming current and future hurdles. Moreover, retinal organoids can provide us with the data needed to advance various fields of research—for example, models of retinal development and models of retinal pathology. Moreover, they can be used as cell sources for transplants and drug screening. However, recapitulation of human retinal development is currently only a dream. Retinal organoids face many constraints; in particular, visual responses are constrained by physiological pathways. Such challenges have stimulated scientists to advance technologies that may one day generate fully functional retinal organoids. Despite all of these limitations, 3D retinal organoids have already offered unprecedented opportunities to understand some of the mechanisms of retinal development, and have shed light on the pathomechanisms of retinal diseases and cell transplantation. We are close to reaching a technological tipping point where 3D retinal organoids will be able to fully address important questions.

## Author Contributions

MF and ZH reviewed the literature, and drafted and edited the manuscript. CR corrected and edited the manuscript. All authors approved the final version of the manuscript.

## Conflict of Interest

The authors declare that the research was conducted in the absence of any commercial or financial relationships that could be construed as a potential conflict of interest.
